# Ceftriaxone-Induced Encephalopathy in a Patient With End-Stage Renal Disease on Hemodialysis

**DOI:** 10.7759/cureus.46226

**Published:** 2023-09-29

**Authors:** Athmananda Nanjundappa, Salina Munankami, Khalid Al Talib, Jyotsna Gummadi, Sumanth Kumar Bandaru

**Affiliations:** 1 Department of Internal Medicine, MedStar Franklin Square Medical Center, Baltimore, USA; 2 Department of General Medicine, Kathmandu Medical College, Kathmandu, NPL; 3 Department of Nephrology, MedStar Franklin Square Medical Center, Baltimore, USA; 4 Department of Internal Medicine, MedStar Harbor Hospital, Baltimore, USA

**Keywords:** prolonged antibiotics, acute metabolic encephalopathy, hemodialysis related, iv ceftriaxone, drug-induced encephalopathy

## Abstract

Ceftriaxone-induced encephalopathy is a rare but known adverse effect secondary to neurotoxicity, especially in patients with end-stage renal disease (ESRD) on hemodialysis. The common presenting symptoms include myoclonus, psychosis, and seizures. We are presenting a case of a 77-year-old female patient who presented with confusion and jerky movements of her lips and extremities. Her initial workup was negative for stroke and seizure disorder. A probable diagnosis of ceftriaxone-induced encephalopathy was made using an Adverse Drug Reaction (ADR) probability scale (Naranjo scale) with a Naranjo score of 5. The patient's symptoms resolved after discontinuation of ceftriaxone. Ceftriaxone is a commonly used intravenous antibiotic in the inpatient setting, and thus clinicians should be aware of this rare adverse reaction in patients with ESRD.

## Introduction

Ceftriaxone is a third-generation bactericidal cephalosporin typically used against Gram-negative aerobic bacilli infection [1]. Ceftriaxone is widely distributed throughout the body, including cerebrospinal fluid (CSF), gallbladder, lungs, and bile [2]. The half-life of ceftriaxone in adults with normal renal and hepatic function is about 5 to 9 hours. However, the half-life can rise to 12 to 16 hours in adults with severe renal impairment [2]. Neurotoxicity is a known but rare adverse reaction of cephalosporins with symptoms including encephalopathy, altered mental status, myoclonus, and seizures, which are believed to be caused due to high levels in the CSF [3]. It is somewhat more common in patients with end-stage renal disease (ESRD) due to impaired excretion, as it is challenging to eliminate serum ceftriaxone by hemodialysis because of the high binding rate of ceftriaxone with serum albumin (90%) [3, 4]. We hereby present a rare case of ceftriaxone-induced encephalopathy in an elderly patient with ESRD on hemodialysis.

## Case presentation

A 77-year-old Caucasian woman with a history of ESRD on hemodialysis thrice a week, hypertension, chronic obstructive pulmonary disease (COPD) on home oxygen 3 l/min and insulin-dependent type 2 diabetes mellitus presented from a skilled nursing facility with confusion and speech disturbance for three days prior to her presentation. Skilled nursing facility staff had reported the patient was ”not herself” for three days. On initial evaluation, the patient had incoherent speech and experienced intermittent jerky movements involving her lips and bilateral upper and lower extremities. 

On reviewing her past medical history, the patient was recently discharged from the same hospital three weeks ago with a diagnosis of *Streptococcus miti*s bacteremia secondary to an infected arteriovenous fistula. She was consulted by an Infectious Disease consultant and started on intravenous (IV) ceftriaxone 2 grams daily for a total duration of six weeks.

In the emergency department, she had a pulse rate of 72/min (regular), non-tachypneic with a respiratory rate of 18/min, hypertensive with a blood pressure of 178/82 mmHg, afebrile with a temperature of 98.6°F and oxygen saturation 97% on oxygen 3 l/min. The patient was awake but not oriented to time, place, or person and was not following commands. She was mumbling incoherent words. The neurological exam was limited due to the inability to follow orders. However, she was moving all her extremities independently. Bilateral pupils were reactive, reflexes brisk in bilateral upper and lower extremities, and clonus was absent. The patient had random myoclonic jerky movements noted especially in her bilateral upper extremities. The rest of the physical exams were unremarkable.

Initial laboratory analysis performed in the emergency department (Table 1) revealed an unremarkable basic metabolic panel. Serum ammonia level, thyroid stimulating hormone (TSH), hepatic function panel, and ionized calcium levels were within normal limits. A complete blood count revealed mild macrocytic anemia, with normal white blood cells and platelet count. A computerized tomographic (CT) scan of the head did not show any acute intracranial abnormalities. A chest X-ray and electrocardiogram (EKG) were unremarkable. Magnetic resonance imaging (MRI) of the brain did not show any cerebral ischemic stroke or hemorrhage.

**Table 1:** Laboratory analysis results on admission HDL, high-density lipoprotein; LDL, low-density lipoprotein; TSH, thyroid-stimulating hormone

Laboratory Variables	Lab Values	Reference Range
Sodium (mmol/L)	138	136-145
Potassium (mmol/L)	4.4	3.5-5.1
Chloride (mmol/L)	100	98-107
CO2 (mmol/L)	34	21-31
Blood urea nitrogen (mg/dL)	52	7-25
Creatinine (mg/dL)	4.27	0.6-1.3
Calcium (mg/dL)	8.8	8.6-10.3
Magnesium (mg/dL)	2.1	1.9-2.7
Phosphorus(mg/dl)	2.8	3.4-4.5
Total protein (g/dL)	6.6	6.4-8.9
Direct bilirubin (mg/dL)	0.1	0.0-0.2
Total bilirubin (mg/dL)	0.2	0.3-1.0
Alkaline phosphatase (IU/L)	91	34-104
Aspartate aminotransferase (IU/L)	14	13-39
Alanine aminotransferase (IU/L)	7	7-52
Anion Gap (mmol/L)	4	5-12
Albumin (g/dL)	3.6	3.5-5.7
Ionized Calcium (mmol/L)	1.19	1.2-1.4
Lipase (IU/L)	27	11-82
Total cholesterol (mg/dL)	151	<200
Triglycerides (mg/dL)	161	<175
HDL cholesterol (mg/dL)	42	23-92
LDL calculated (mg/dL)	77	<100 Optimal
Glucose (mg/dL)	169	70-99
Ammonia (µ/dL)	10	15-45
TSH (uIU/mL)	2.518	0.45-5.33
White blood cell count (x103/µl)	8.2	4.8-10.8
Red blood cell count (x106/µl)	2.95	4.5-6.1
Hemoglobin (g/dL)	9.0	14-17.5
Hematocrit (%)	30.2	39-53
Platelet count (x103/µl)	311	130-400
Mean Corpuscular Volume (fL)	102.4	80-100
Mean Corpuscular Hemoglobin (pg)	30.5	26-33
Red Cell Distribution Width (%)	16	12-15

The patient was hospitalized under the Internal Medicine team for further evaluation of her confusional state and myoclonic movements of her limbs. The patient underwent a magnetic resonance imaging (MRI) test that was negative for any infarcts or hemorrhages. Her presentation is clinically consistent with acute toxic-metabolic encephalopathy (TME) given the acute confusional state with features of sympathetic hyperactivity such as myoclonic jerks and tremors. Common causes of TME, such as electrolyte disorders, primary structural brain disease, and impaired oxygen delivery, were ruled out. The patient was consulted by a Nephrologist and underwent hemodialysis, however, her symptoms persisted. An electroencephalogram (EEG) performed revealed mild to moderate diffuse slowing, which is a non-specific finding suggesting of diffuse or bilateral cerebral dysfunction. 

Since the patient was on long-term antibiotic therapy with IV Ceftriaxone for a recent diagnosis of *Streptococcus mitis* bacteremia, the patient was suspected of ceftriaxone-induced encephalopathy. Unfortunately, our hospital laboratory was unable to perform plasma ceftriaxone levels. After discussions with the Infectious Disease consultant, ceftriaxone was discontinued and replaced with IV vancomycin for the treatment of *Streptococcus mitis* bacteremia. The patient’s confusion and myoclonic jerking movements improved and resolved over the next 2 days. An Adverse Drug Reaction (ADR) probability scale (Naranjo scale) was used to assess the causal relationship between ceftriaxone use and the encephalopathy. With a Naranjo score of 5, we were able to establish a probable diagnosis of ceftriaxone-induced encephalopathy in our case [5]*.* The patient was continued on IV vancomycin and eventually completed a total of 6 weeks of antibiotic therapy.

## Discussion

Encephalopathy is an infrequent ceftriaxone adverse effect and is assumed to be caused by competitive antagonism of brain gamma-aminobutyric acid in the central nervous system and increased excitatory amino acid levels due to high levels of ceftriaxone in CSF [6]. Most cases involve renal impairment, and more than half of these patients are on hemodialysis or peritoneal dialysis [7]. However, unlike most cephalosporins, ceftriaxone is not dialyzed during hemodialysis and can accumulate in patients with severely impaired renal function [8]. The usual dose of ceftriaxone is 1-2 g every 12 to 24 hours [2]. Dosage adjustment is generally not recommended in renal impairment unless concurrent hepatic dysfunction is present with a maximum dose less than or equal to 2000 mg/day [2]. As ceftriaxone is eliminated via urinary and biliary excretion, symptoms usually start a week after the administration of ceftriaxone and show results within one or two weeks after the dissolution of the drug [9]. Clinical features include psychosis, seizure, myoclonus, and choreoathetosis [3]. Usually, laboratory analysis and imaging, including a computerized scan (CT) scan of the head and magnetic resonance imaging (MRI) of the head, are unremarkable. However, an electroencephalogram (EEG) can show slow and generalized periodic discharge with triphasic morphology [10]. Few cases have cited the association between high blood and CSF ceftriaxone levels with encephalopathy [11]. Treatment includes the discontinuation of ceftriaxone and hemoperfusion in severe patients [12]. Most of the cases that have been reported support the diagnosis based on the recent history of ceftriaxone exposure, improvement after ceftriaxone discontinuation, and exclusion of other encephalopathy causes [7]. Most cases that have been reported support the diagnosis based on recent history of ceftriaxone exposure, improvement after ceftriaxone discontinuation, and exclusion of other encephalopathy causes [7].

## Conclusions

Ceftriaxone-induced encephalopathy is a rare but known complication in ESRD patients on hemodialysis, as the drug is non-dialyzable. Being one of the most common IV antibiotics to be used in inpatient settings, clinicians should be aware of this adverse reaction. Therefore, even though no dosage adjustment is recommended in ESRD, physicians should consider ceftriaxone as a cause of encephalopathy in ESRD patients, especially those treated for more than a week, and when other causes of encephalopathy have been ruled out.
